# Synergistic effect of the TLR5 agonist CBLB502 and its downstream effector IL-22 against liver injury

**DOI:** 10.1038/s41419-021-03654-3

**Published:** 2021-04-06

**Authors:** Nicolas Melin, Daniel Sánchez-Taltavull, René Fahrner, Adrian Keogh, Michel Dosch, Isabel Büchi, Yitzhak Zimmer, Michaela Medová, Guido Beldi, Daniel M. Aebersold, Daniel Candinas, Deborah Stroka

**Affiliations:** 1 Department for BioMedical Research, Inselspital, Bern University Hospital, University of Bern, 3008 Bern, Switzerland; 2 Visceral Surgery and Medicine, Inselspital, Bern University Hospital, University of Bern, 3008 Bern, Switzerland; 3 Department of Radiation Oncology, Inselspital, Bern University Hospital, University of Bern, 3010 Bern, Switzerland; 4 Present Address: Department of General, Visceral and Vascular Surgery, Bürgerspital Solothurn, 4500 Solothurn, Switzerland

**Keywords:** Liver diseases, Acute inflammation

## Abstract

The toll-like receptor 5 (TLR5) agonist, CBLB502/Entolimod, is a peptide derived from bacterial flagellin and has been shown to protect against radiation-induced tissue damage in animal models. Here we investigated the protective mechanism of CBLB502 in the liver using models of ischemia-reperfusion injury and concanavalin A (ConA) induced immuno-hepatitis. We report that pretreatment of mice with CBLB502 provoked a concomitant activation of NF-κB and STAT3 signaling in the liver and reduced hepatic damage in both models. To understand the underlying mechanism, we screened for cytokines in the serum of CBLB502 treated animals and detected high levels of IL-22. There was no transcriptional upregulation of IL-22 in the liver, rather it was found in extrahepatic tissues, mainly the colon, mesenteric lymph nodes (MLN), and spleen. RNA-seq analysis on isolated hepatocytes demonstrated that the concomitant activation of NF-κB signaling by CBLB502 and STAT3 signaling by IL-22 produced a synergistic cytoprotective transcriptional signature. In IL-22 knockout mice, the loss of IL-22 resulted in a decrease of hepatic STAT3 activation, a reduction in the cytoprotective signature, and a loss of hepatoprotection following ischemia-reperfusion-induced liver injury. Taken together, these findings suggest that CBLB502 protects the liver by increasing hepatocyte resistance to acute liver injury through the cooperation of TLR5-NF-κB and IL-22-STAT3 signaling pathways.

## Introduction

Hepatic diseases are a major health care burden worldwide. In many cases, the necessary treatment option for end-stage hepatic disease is liver transplantation, however, less than 10% of the global transplantation needs are currently met^1^. New methods of providing protection against hepatic injury, thus ameliorating continued liver dysfunction and failure, may provide support for this shortcoming.

The innate immune system recognizes molecules shared by pathogens through germ-line encoded toll-like receptors (TLR). When activated, TLRs recruit toll/interleukin-1 receptor (TIR) domain-containing adapter proteins such as myeloid differentiation primary response 88 (MyD88) and TIR-domain-containing adapter-inducing interferon-β (TRIF), which initiate signal transduction pathways that culminate in the activation of nuclear factor kappa B (NF-κB), interferon regulatory factors (IRF), or mitogen-activated protein kinases (MAPK)^2^. Regulation of TLR signaling is important as its sustained activation can exacerbate an inflammatory response resulting in cellular and tissue damage^2^, such as the amplification of inflammation in rheumatoid arthritis^3^, inflammatory bowel disease^4^, acute injury provoked by ischemia-reperfusion^5^, and tissue fibrosis^6^. On the other hand, judicious activation of TLRs can be beneficial for tissue repair and protection by activating cytoprotective, anti-inflammatory signaling pathways^6,7^.

TLR5 is activated via the engagement of leucine-rich repeat (LRR) ectodomains of TLR5 with its natural ligand, the three helices of the flagellin D1 domain^8^. TLR5 agonists were shown to limit ischemia-reperfusion injury (IRI) in the kidney^9^ and to decrease graft versus host disease^10^. The beneficial effect of TLR5 activation has been intensely investigated in the context of radiation-induced toxicity, resulting in the development of the optimized flagellin derivative, Entolimod, or CBLB502 (CBLB)^11^. Its proposed mechanism of protection is the increased survival of intestinal and hematopoietic stem cells by reducing inflammation and promoting antiapoptotic and redox modulating pathways^12^. Based on its immunostimulatory effect and protective properties, the use of CBLB has advanced towards clinical trials to promote antitumor responses, to reduce radiotherapy and/or chemotherapy-induced side effects, and to be used as a potentiating adjuvant during vaccinations (www.clinicaltrial.gov. trial ref: NCT02715882, NCT03063736, NCT01527136, NCT01728480).

Based on the studies demonstrating its protective activity in normal tissue, we investigated the mechanism of CBLB’s protection in the liver. We used two hepatic injury models; IRI as a ROS and immune-induced hepatic damage model^13^, and autoimmune-hepatitis induced by concanavalin A (ConA) as a hepatocyte-apoptosis model. Our results demonstrate that CBLB protects against hepatic damage by protecting against parenchymal cell death. The hepatoprotective effect is not solely achieved by TLR5 activation on hepatocytes but requires concomitant signal transducer and activator of transcription 3 (STAT3) signaling induced by remote CBLB-mediated IL-22 secretion.

## Materials and Methods

### Animal models

Animals were housed in specific pathogen‐free conditions in accord with the Swiss veterinary office. Eight to twelve weeks old C57BL/6J male and female mice (Harlan Laboratories B.V., Horst, Netherlands), MyD88/TRIF double knockout mice^14^, and IL-22 knockout mice^15^ were acclimatized in a temperature‐controlled room with a 12 hours dark/light cycle and ad libitum access to water and chow. CBLB was dissolved in saline and injected i.p. (5μ l/g mouse) to reach a final concentration of 0.2 mg/kg CBLB or an equivalent volume of saline (vehicle) in a randomized (equal distribution of animals amongst cages) and blinded (CBLB vs. Saline) 2 h prior to the IRI or ConA challenge. IRI was induced by depriving the left lobe of blood inflow for 1 h according to the previously described procedure^16^. Serum was collected 6 h post-reperfusion and mice were sacrificed at 24 h. Tissue from the left lobe was fixed in 4% PFA for 48 h and snap-frozen. ConA injury was provoked following a published protocol^17^ with 12 mg/kg of ConA injected i.v. (Sigma-Aldrich, Darmstadt, Germany, #C7642). Mice were sacrificed after 8 h, serum was collected, the left liver lobe was fixed in 4% PFA for 24 h, and the right lobe was snap frozen. Animal procedures were approved by the Veterinary Office of the Canton Bern in line with institutional and standard protocols for the care and use of laboratory animals.

### Measurement of serum alanine transaminase

Post‐injury serum samples were collected to assess the extent of liver injury using alanine transaminase assay (P800; Modular Analytics EVO, Roche, Mannheim, Germany). The results were analyzed and displayed with GraphPad Prism software, representing the mean and standard deviation and analyzed using two-sided Student’s *t*-test. (GraphPad Software Inc., La Jolla, CA, USA).

### Histology

Hematoxylin and eosin staining and Gr1 immunohistochemistry (eBioscience, San Diego, CA, USA, 4-5931-85) were done following standard protocols previously described^16^ and imaged with the Panoramic^®^ 250 Flash III slide scanner (3DHISTECH, Budapest, Hungary) using a 20x objective and the #DHISTECH acquisition software at room temperature.

### Myeloperoxidase assay

Proteins were extracted from total liver tissue and myeloperoxidase (MPO) activity assay was carried out as previously described^16^. The results were analyzed and displayed with GraphPad Prism software, representing the mean and standard deviation and analyzed using two-sided Student’s *t*-test. (GraphPad Software Inc., La Jolla, CA, USA).

### Flow cytometry

Liver leukocytes were isolated by mechanical dissociation as described previously^18^. The following fluorescently-labeled antibodies targeting mouse antigens were used: CD45-BUV (BD Biosciences, Franklin Lakes, NJ, USA, 30-F11), CD3-FITC (BioLegend, San Diego, CA, USA, 17A2), NK1.1-APC-eFluor780 (eBioscience, PK136), CD25-APC (ebioscience, PC61.5), and CD69-PeCy7 (BD Biosciences, H1.2F3). Cells were acquired on a SORP LSRII (BD Pharmingen Inc., San Diego, CA) and flow cytometry data were analyzed using FlowJo software (FlowJo, LLC) following the gating strategy given in Supplementary Fig. 1. The results were analyzed and displayed with GraphPad Prism software, representing the mean and standard deviation and analyzed using two-sided Student’s *t*-test. (GraphPad Software Inc., La Jolla, CA, USA).

### Serum cytokine measurements

Circulating cytokines and chemokines were assessed using the magnetic bead array based ProcartaPlex mouse basic kit (#EXP010-20440-901) in combination with the targets kit (Supplementary Table 1), following the manufacturer’s instructions (eBioscience) and measured using Luminex instrument (xMAP Technology). The results were analyzed and displayed with GraphPad Prism software, representing the mean and standard deviation. (GraphPad Software Inc., La Jolla, CA, USA).

### RNA isolation and quantification

RNA was isolated from snap-frozen tissue using TRIzol (Sigma), quantified, and cDNA was generated using Omniscript RT Kit 200 (Qiagen, Hilden, Germany) as previously described^19^. qPCRs were performed using TaqMan gene expression assays for Il22, Tbp, Socs3, Hmox1, and Tnfaip3 (Thermo Fisher Scientific; Supplementary Table 2). Il22 RNA was quantified following the absolute quantification methods described by Pfaffl et al.^20^. Tbp PCR product was cloned in a pGEM^®^-T Easy Vector (Promega, Madison, WI, USA, #A3600), the plasmid was amplified using the One Shot TOP10 Chemically Competent *E. coli* (Thermo Fisher Scientific) and purified using the PureYield MaxiPrep (Promega). Il22 was quantified based on the Tbp standard, assuming a perfect PCR efficacy. For the other targets, relative changes in mRNA were calculated with the ΔΔCt method using Tbp as a control gene. The results were analyzed and displayed with GraphPad Prism software, representing the mean and standard deviation and analyzed using two-sided Student’s *t*-test. (GraphPad Software Inc., La Jolla, CA, USA).

### Western blot

Western blots were done following previously used protocols^21,22^ and the primary antibody for IκBα (Santa Cruz Biotechnology, Dallas, TX, USA #sc371), and STAT3 (#9139), phospho-STAT3-Tyr705 (#9145) or TBP (#8415) all from Cell Signaling Technology (Danvers, MA, USA), were used at a 1:1000 dilution, and the secondary antibodies were anti-rabbit-HRP (Dako, Agilent technology, Santa Clara, CA, USA) or anti-rabbit-680LT or anti-mouse-800CW (LI-COR Bioscience, Lincoln, NE, USA). Band densities were analyzed using ImageJ and displayed with GraphPad Prism software, representing the mean and standard deviation and analyzed using two-sided Student’s *t*-test. (GraphPad Software Inc., La Jolla, CA, USA).

### Hepatocyte isolation and culture

C57BL/6 mouse hepatocytes were isolated in situ with collagenase perfusion as previously described^23^, and cultured on rat tail collagen coated plate in arginine free Williams E media supplemented with 40 mM ornithine, 100 iU/ml insulin, 2 mg/ml hydrocortisone, penicillin, and streptomycin. Twenty-four hours after plating, the hepatocytes were treated with CBLB (4 ng/ml Cleveland BioLabs, Buffalo, NY, USA) and/or IL-22 (1 ng/ml; PeproTech, Rocky Hill, NJ, USA, #210-22) in combination.

### Transcriptomic and pathway analysis

RNA was extracted from snap-frozen tissue with RNeasy Micro Kit (Qiagen; 74004), quantified by Qubit BR assay (Thermo Fisher Scientific) and quality assessed by Fragment Analyzer (Advanced Analytical, Parkersburg, WV, USA) using Standard Sensitivity RNA Analysis Kit (Advanced Analytical, #DNF-471). The library was prepared without size exclusion with the TruSeq Stranded mRNA kit (Illumina, San Diego, CA, USA, #20020594). Qubit dsDNA HS (Thermo Fisher Scientific; #Q32851) and Fragment Analyzer NGS Kit (Advanced Analytical; #DNF-474) were used for library quality control. Finally, the samples were sequenced on a NovaSeq6000 (Illumina), paired-end 1 x 50 bp on one lane. Fastq files were aligned to the mouse reference genome mm10 with HISAT2^24^. The resulting SAM files were transformed into sorted BAM files with SAMTOOLS^25^. Further analysis was done using the RStudio software

The counts were counted from the bam files with featureCounts R function of the R package Rsubread^26^. Differentially expressed genes (DEG) were assessed using DESeq2^27^. In every condition (control, CBLB, IL-22, CBLB + IL-22), a paired comparison within each isolation replicate was performed. In order to visualize the data in two dimensions, we transformed the data into reads per million (RPM) and performed a principal component analysis with the R function prcomp. The new resulting coordinates (PC1 and PC2) were translated to move the Control to the position 0, 0, that is f_i_(x,y) = (x_i_ − c_x,i,_ y_n_ − c_y,i_), with (i = 1, 2, 3) denoting the hepatocytes isolation experiment. Gene Log10(*p* value) and Log2(fold change) (log2FC) changes for each condition were visualized using volcano plots with the gene with an adjusted *p* value < 0.05 and −1 < Log2FC > 1 were colored in green. A heatmap figure was produced using normalized gene expression with the maximum expression set to 1 and the minimum to 0, using the function *heatmap.2* from the R package^28^, with complete linkage using correlation distance.

The stackplots showing the pathways were performed with a custom R script. First, we selected the ten most significant families of pathways identified by Metascape. Second, we obtained all the genes involved with the R function gconvert of the R package gprofiler2. Third, we computed the intersection between the gene lists and the statistically significant differentially expressed and colored according to log2FC. The significance of each pathway was assessed by Metascape. For the p53 signaling pathway (mmu04115), the genes were manually included from the Metascape output. The bar plots showing the 20 most significant families of pathways were done with Metascape.

## Results

### TLR5 agonist CBLB is hepatoprotective in vivo

**Fig. 1:**
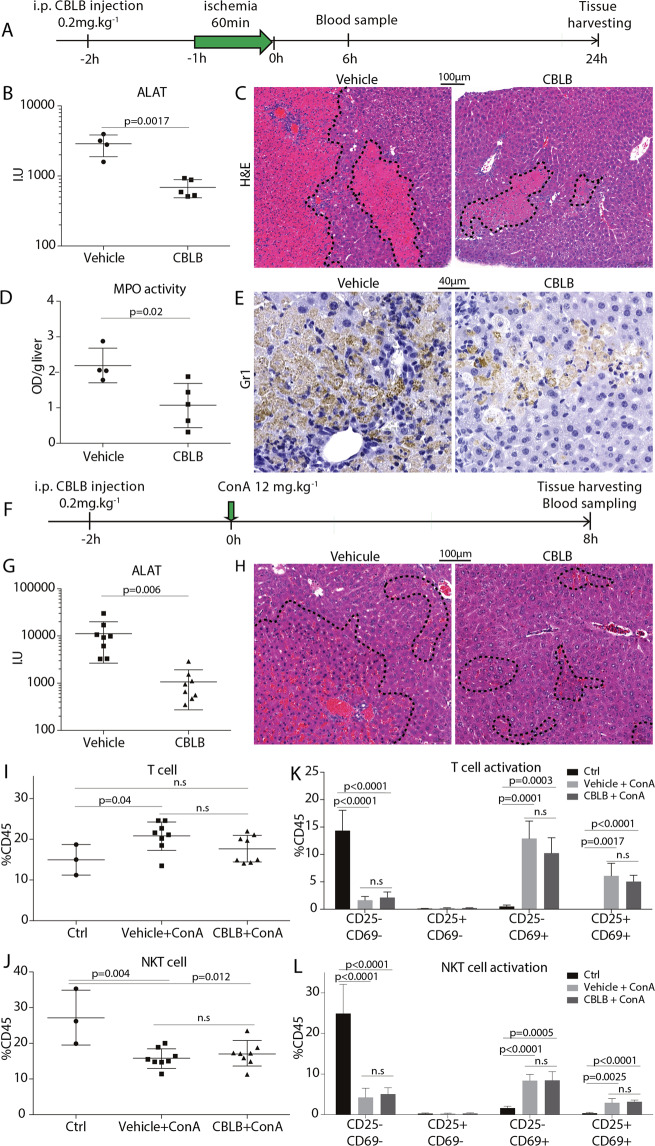
CBLB decreases hepatic damage. **A** Experimental plan of ischemia-reperfusion study, vehicle *n* = 4, CBLB *n* = 5 mice. **B** Serum ALAT 6 h after reperfusion. **C** Representative H&E image of liver tissue 24 h post-reperfusion. **D** Tissue myeloperoxidase activity 24 h post-reperfusion. **E** Representative images of liver Gr1 immunohistochemistry 24 h post-ischemia-reperfusion. **F** Experimental plan of ConA model, vehicule *n* = 8, CBLB *n* = 8. **G** Serum ALAT 8 h after ConA challenge. **H** Representative H&E image of liver tissue 8 h following ConA injection. FACS analysis of **I** T cell and **J** NKT cells frequency represented as a percentage of CD45 + cells extracted from the liver 8 h ConA injection. FACS analysis of the activation frequency of **K** T cell and **L** NKT cell markers CD25 and CD69. Frequencies are represented as a percentage of CD45 + cells extracted from liver 8 h post-ConA injection.

The effect of CBLB against oxidative stress generated by ischemia-reperfusion and by immune-driven hepatic damage caused by ConA was tested in mice pretreated with CBLB for 2 h (Fig. 1A, F). Following 1 h of partial liver ischemia and 6 h reperfusion, serum ALAT levels elevated to 2883I.U. in control animals, whereas ALAT levels only increased to 688I.U. in animals pretreated with CBLB (Fig. 1B). Twenty-four hours after reperfusion, the livers of CBLB treated mice displayed smaller necrotic areas (Fig. 1C), which was accompanied by a reduction of MPO activity (Fig. 1D) resulting from the decreased neutrophil infiltration (Fig. 1E). In the ConA model, CBLB pretreated animals had a tenfold decrease of ALAT serum levels (from 11186I.U. to 1068I.U.) following ConA challenge compared to control (Fig. 1G), and a decrease in necrotic areas compared to controls (Fig. 1H). T cells and NKT cells are the primary effector cells in mediating ConA injury^17,29^. In control mice treated with ConA, there was an increase in the percentage of T cells in the infiltrating CD45+ cells (Fig. 1I), and a reduced percentage of NKT cells (Fig. 1J). In both T and NKT cells, there was an increase in their activation markers CD25 and CD69, with more double-positive cells present (Fig. 1K, L). CBLB did not alter the percentage of infiltrating immune cells nor did it modify their activation marker expression upon ConA challenge (Fig. 1I–L). Taken together, our in vivo data suggests that pretreatment with CBLB prevents hepatic damage in both IRI and ConA liver injury, as determined by the early reduction in serum ALAT levels. As there was no difference in the NKT and T cell infiltration and activation in the ConA model, this suggested that CBLB did not alter the immune cell infiltration and activation, but rather may enhance the resistance of hepatocytes to harmful stimuli.

### CBLB induces hepatic STAT3 activation and remote IL-22 production

**Fig. 2:**
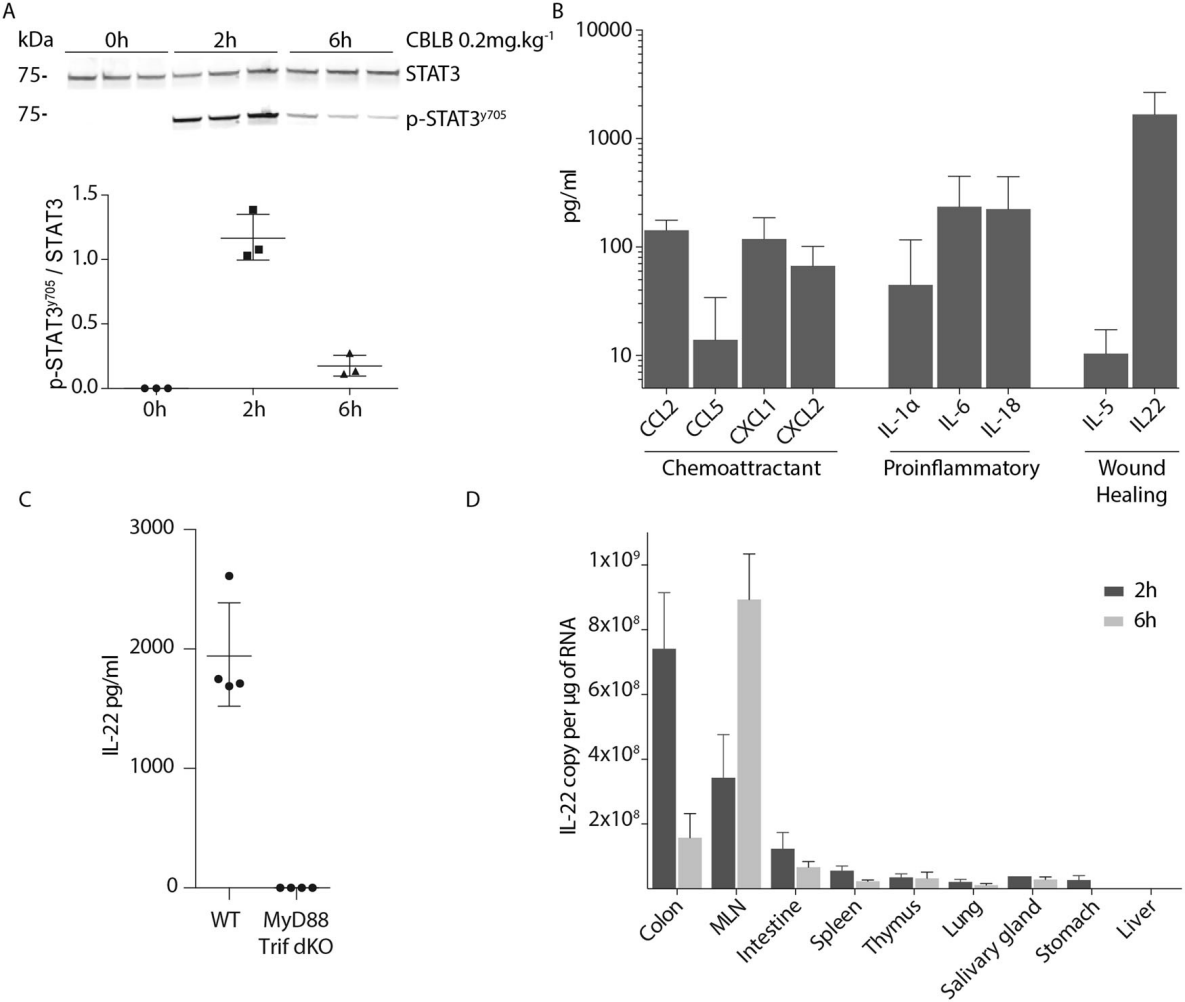
CBLB induces a remote IL-22 release that triggers hepatic STAT3 signaling. **A** Western blot analysis of liver STAT3 and STAT3 phosphorylated at tyrosine 705 at control, 2 h, and 6 h following CBLB injection, and its STAT3 phosphorylated at tyrosine 705/STAT3 ratio, *n* = 3 mice per group. **B** Analysis of mouse serum 2 h post-CBLB injection, *n* = 7 mice. **C** IL-22 serum levels in WT and MyD88/Trif double knockout mice 2 h post-CBLB injection, *n* = 4 mice per group. **D** RNA expression in various tissue 2 and 6 h post-CBLB injection, *n* = 3 mice per group.

Previous reports have shown that CBLB activates both NF-κB and STAT3 signaling pathways in vivo^12^. As CBLB is known to directly activate the NF-κB pathway via TLR5 signaling^30^, we explored the origin of the CBLB-induced STAT3 activation in the liver. Two hours post-injection, CBLB induced the phosphorylation of tyrosine 705 of STAT3 in liver tissue (Fig. 2A) As STAT3 signaling is mainly mediated by Janus kinases upon cytokine stimulation^31^, we evaluated the serum of mice for 22 cytokines and chemokines 2 and 6 h following CBLB exposure. TLR5 activation did not increase the serum level of 13 of the tested cytokines, IL-1β, IL-4, IL-10, IL-12p70, IL-13, IL-15/IL-15R, IL-17A, IL-17F, IL-23, IL-33, TNFα, INFα, and INFγ (Supplementary Fig. 2), whereas nine other cytokines were elevated in the serum with a peak 2 h post-injection. There was a minor increase of the chemokine CCL5 and a greater than tenfold increase of CCL2, CXCL1, and CXCL2 to ~100 pg/ml. The pro-inflammatory cytokine IL-1α was increased to 50 pg/ml and IL-6 and IL-18 were increased to around 200 pg/ml. For cytokines involved in wound healing, IL-5 showed a minor response, whereas serum levels of IL-22 reached an average level of 1670 pg/ml (Fig. 2B). IL-22 is an anti-fibrotic cytokine, that prevents liver injury, promotes liver regeneration^32,33^, and was shown to protect against both hepatic IRI and ConA-induced liver damage^34,35^. Consequently, we considered IL-22 as a possible contributor to the hepatoprotective effect of TLR5 activation.

To assess if the increase of IL-22 in the serum of CBLB treated mice was due to TLR5 signaling, CBLB was administered to MyD88/TRIF double KO mice. There was no increase in serum IL-22 levels, suggesting that CBLB increased levels of IL-22 in a TLR5-MyD88-TRIF-dependent manner (Fig. 2C). We next questioned if the increase of IL-22 serum levels was a direct and localized response from the liver. We observed no transcriptional increase of IL-22 in the liver tissue of mice 2 and 6 h following CBLB injection (Fig. 2D). To then determine the source of IL-22 detected in serum, we screened 17 organs for IL-22 mRNA expression following CBLB exposure. There were no detectable levels of IL-22 mRNA in all organs prior to CBLB injection, nor in the pancreas, bone marrow, skin, kidney, brain, muscle, heart, and blood 2 h post-injection (Supplementary Fig. 3). In the colon and MLN, there was a strong increase in IL-22 mRNA, and to a smaller extent also in the intestine, spleen, thymus, lung, salivary gland, and stomach. After 6 h, IL-22 mRNA levels were lower in all organs, with the exception of the MLN that showed a 2.5 times higher level of IL-22 transcript compared to values at 2 h (Fig. 2D and Supplementary Fig. 3). Taken together, our data demonstrate that in addition to NF-κB activation, STAT3 is activated in the liver of mice exposed to CBLB. STAT3 activation can be a result of TLR5-induced cytokine expression, particularly IL-22. We furthermore conclude that IL-22 is not produced in the liver, but rather remotely in the colon and MLN.

### Pathway analysis of hepatocytes stimulated with CBLB and IL-22

**Fig. 3:**
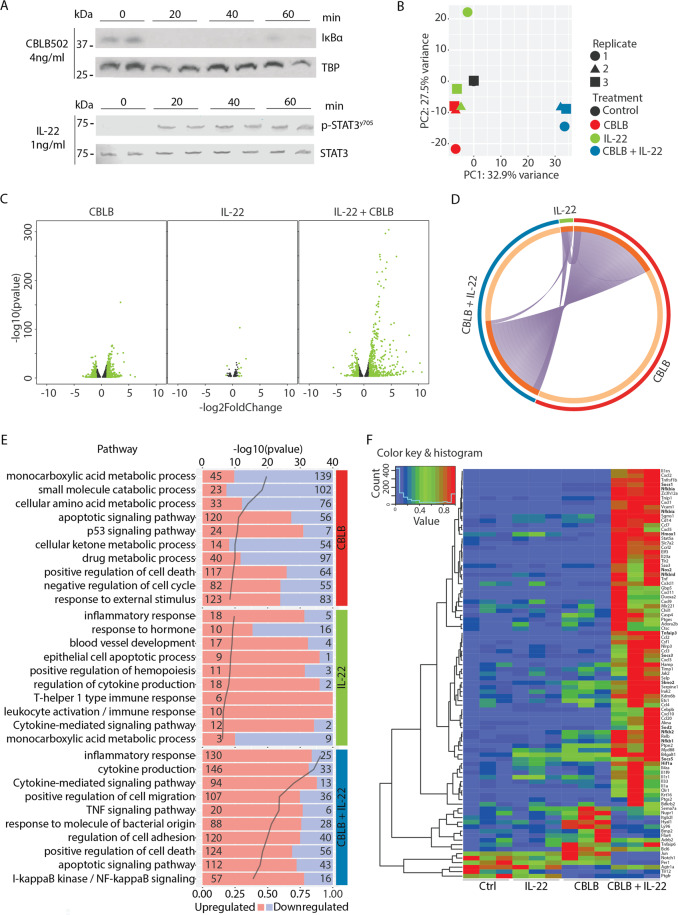
CBLB and Il-22 induce hepatocyte NF-κB and STAT3 signaling, respectively. **A** Western blot analysis of hepatocytes for IκBα normalized by TBP and STAT3 phosphorylated at tyrosine 705 normalized by STAT3 at control, 20, 40, and 60 min after CBLB or IL-22 exposure, *n* = 2 replicates per time point. **B** The PCA of the transcriptomic signature of hepatocytes treated for 2 h with either CBLB in red, IL-22 in green, CBLB + IL-22 in blue, or control in black. The isolation replicates are indicated with the same shape, *n* = 3 replicates. **C** Volcano plot displaying the differentially expressed genes (DEG) of each condition compared to control, showing the DEG with a −1 < Log2FC > 1 in green. **D** Circos plot showing on the outer ring the treatment groups and on the inner ring the DEG. DEG that are shared by the different groups are indicated in dark orange, DEG unique to each group are in light orange. Each gene shared between the different groups is indicated by purple lines. **E** Bar graph presenting the ten most regulated pathways of each condition, with the number of DEG upregulated in red or downregulated in blue for each condition. **F** Heat map of the inflammatory response pathway showing the DEG with a −1 < Log2FC > 1, with genes known to be hepatoprotective in IRI or ConA models in bold.

We next questioned if CBLB and/or IL-22 directly alter the transcriptional profile of hepatocytes thus explaining its hepatoprotective effect. We first validated that hepatocytes have a direct response to TLR5 activation and IL-22 by treating primary mouse hepatocytes with CBLB and IL-22 and measuring downstream receptor signaling. CBLB led to the activation of the NF-κB, indicated by the degradation of its inhibitory protein IκBα but did not lead to STAT3 phosphorylation (Fig. 3A and Supplementary Fig. 4). IL-22 induced the phosphorylation of STAT3 on tyrosine 705, signifying activation of STAT3 transcriptional activity but did not result in the activation of NF-κB (Fig. 3A and Supplementary Fig. 4). The downstream transcriptional response was measured by bulk RNA-seq in hepatocytes isolated from three mice that were treated with either CBLB or IL-22 alone or in combination (CBLB + IL-22) for 2 h. The first principal component (PC1) indicated a strong combinatorial effect of CBLB + IL-22, which clustered to the right compared to the control, IL-22 and CBLB treated cells, clustered to the left. In the second principal component (PC2), differences between control and CBLB groups could be observed (Fig. 3B). We next assessed the distribution of statistically significant DEG in the three treatment conditions compared to their untreated controls. CBLB treated hepatocytes displayed 1771 downregulated and 2045 upregulated DEG. The effect of IL-22 on hepatocytes was minor; only 52 downregulated and 100 upregulated DEG were observed. The combination of CBLB + IL-22 resulted in 1184 downregulated and 1587 upregulated DEG. Although the exposure to IL-22 only induced a small effect, adding IL-22 to CBLB altered the response to CBLB alone. Indeed, the addition of IL-22 to CBLB decreased the overall number of DEG and considerably increased their fold change intensity (Fig. 3C).

Next, we questioned if the changes in the DEG between the groups were the same. Gene overlap analysis was performed using the multiple gene list function on Metascape and was then displayed in a Circos plot. IL-22 had no unique DEG; all were shared with the CBLB + IL-22 group. While CBLB and CBLB + IL-22 had shared DEG, both also had a majority of non-overlapping, unique DEG (Fig. 3D) which is further displayed as a heatmap in Supplementary Fig. 5.

To evaluate the functional consequences elicited by each treatment, we performed a pathway enrichment analysis using Metascape which generated a list of 40 pathways associated with up- and down-regulated DEG for each condition (Supplementary Fig. 6). Based on *p* values ranging from −log(*p*) 8.4 to 19.6, the ten most regulated pathways in hepatocytes treated with CBLB alone were comprised of downregulated genes involved in metabolic processes, and upregulated genes are involved in apoptotic signaling and cell cycle regulation. Interestingly, aside from the upregulation of genes involved in the TNF signaling pathway, we did not identify the regulation of genes involved in inflammatory, cytokine, or immune response pathways (Fig. 3E and Supplementary Fig. 6). Pathway enrichment analysis of DEG in IL-22 treated hepatocytes resulted in pathways with −log(*p*) values below 10. The ten most regulated pathways showed an upregulation of genes associated with inflammation, cytokine and immune response, and a downregulation of genes associated with metabolism (Fig. 3E and Supplementary Fig. 6).

The most significantly regulated pathways, with *p* values ranging from −log(*p*) 15.5 to 36.2 were found in the CBLB + IL-22 group and were associated with the upregulation of genes involved in NF-κB and apoptotic signaling, as well as inflammatory, cytokine and immune response pathways. The −log(*p*) values of the pathways associated with downregulated genes in the CBLB + IL-22 group were all below 9.5. (Fig. 3E and Supplementary Fig. 6).

We further investigated the pathway with the highest *p* value across all groups, which was the inflammatory response, and computed a heat map for all the DEG with a fold change below 0.5 or above 2. Strikingly, the combination of CBLB + IL-22 resulted in the upregulation of a large gene set (Fig. 3F). Among those genes strongly upregulated by CBLB + IL-22, we found inhibitors of inflammatory processes such as Nfkbia, Nfkbid, Nfkbiz, Sbno2, Socs1, Socs3, Socs5, and Tnfaip3, and genes regulating redox homeostasis like Hif1a, Hmox1, Nos2, and Sod2, and antiapoptotic genes Tnfaip3 (Supplementary Figs. 7 and 8). The transcriptomic analysis suggests that the combination of CBLB + IL-22 incites a cytoprotective signature that is not accomplished by either of the two treatments alone.

### IL-22 is required for CBLB-induced hepatoprotection

**Fig. 4:**
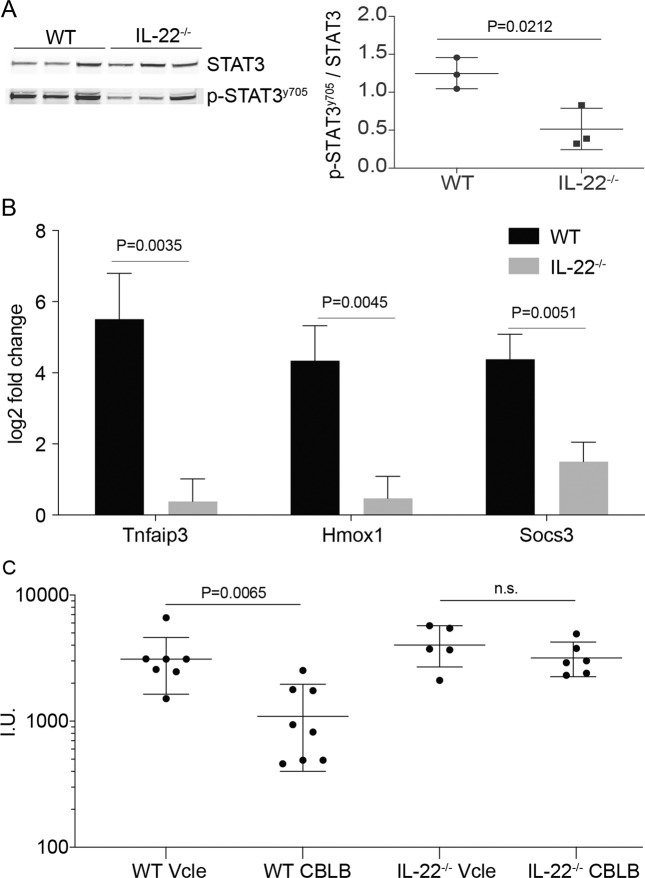
IL-22 is required to mediate CBLB hepatoprotection. **A** Western blot analysis of proteins extracted from the livers of WT and IL-22^−/−^ mice for total STAT3 and STAT3 phosphorylated at tyrosine 705, 2 h following CBLB injection and the corresponding STAT3 phosphorylated at tyrosine 705/STAT3 ratio, *n* = 3 mice per group. **B** qPCR of three of the cytoprotective genes (Tnfaip3, Hmox1, and Socs3) in WT and IL-22^−/−^ mice 2 h following CBLB injection, *n* = 3 mice per group. **C** Serum ALAT levels 6 h after reperfusion.

From the data presented above, IL-22 appeared to have a key role in CBLB-mediated hepatoprotection. For further validation, we assessed the hepatoprotective effect of CBLB in IL-22 knockout (IL-22^−/−^) mice. Firstly, we examined STAT3 activation in the livers of mice following CBLB injection and observed a reduction of STAT3 phosphorylation in IL-22^−/−^ mice (Fig. 4A). Furthermore, the livers of IL-22^−/−^ mice had an attenuated transcriptional increase of the cytoprotective genes, Tnfaip3, Hmox1, and Socs3 in response to CBLB compared to wild type controls (Fig. 4B). Finally, compared to wild-type animals in which CBLB significantly decreased ALAT levels following ischemia-reperfusion, this hepatoprotective effect of CBLB was diminished in IL-22^−/−^ animals (Fig. 4C). Altogether our data suggest that IL-22 is contributing to CBLB-mediated hepatoprotection.

## Discussion

We show that preconditioning mice with the TLR5 agonist CBLB protected them from ischemia-reperfusion and ConA-induced liver injuries. Previous reports support the protective activity of CBLB in models of renal injury and immune-mediated hepatitis, but they focused on the immunomodulatory effect of CBLB, for example, its ability to dampen the immune response^9,36^. In our models, we did not observe an altered immune reactivity in mice pretreated for 2 h with CBLB, namely in NKT and T-cell activation markers, thus suggesting a potential increased resistance within the hepatocytes themselves to injurious stimuli. In agreement with our data, both NF-κB and STAT3 signaling and have been demonstrated to be activated in the liver of mice treated with CBLB^12^. As there is no evidence to directly link TLR5 with STAT3 activation, we investigated an indirect effect which could trigger STAT3 activation by focusing on a CBLB-induced cytokine and chemokine response. Our results show an increase in serum levels of chemoattractant (CCL2, CCL5, CXCL1, and CXCL2), pro-inflammatory (IL-1α, IL-6, and IL-18), and wound healing associated-cytokines (IL-5, IL-22), which is in agreement with the current knowledge of a cytokine response expected following exposure to TLR5 agonists^37–41^. Notably, levels of IL-22 were the highest amongst the cytokines measured. IL-22 signals through IL-22Rα and IL-10Rβ, triggering STAT3 signaling and has been reported to promote liver regeneration and to decrease liver fibrosis and injury^32–35^. We demonstrated that IL-22 secreted after CBLB treatment was not originating from the liver, but from extrahepatic sources, namely organs upstream of the portal vein, such as colon, MLN, spleen, and intestine. This finding is strengthened by the identification of the IL-22 producing cells as CD3^−^, CD127^+^ immune cells from the spleen and mucosa in response to TLR5 activation^38^. Based on these observations, we suppose that IL-22 produced by those visceral organs, circulates via the portal blood flow into the liver and triggers the activation of STAT3 signaling.

To better understand how CBLB could increase the resistance of hepatocytes to damage, we evaluated the effect of CBLB and IL-22 alone or in combination in isolated mouse hepatocytes. As expected, CBLB led to the activation of the NF-κB pathway and resulted in a robust transcriptional response. Interestingly, the majority of the pathways affected were associated with the downregulation of genes associated with metabolism. Recent work reporting a hepatocyte-specific deletion of the TLR downstream adapter protein MyD88, also demonstrated an alteration of hepatic bile acid, glucose, and lipid metabolic functions, supporting our observations^42^. Although we expected to observe a strong inflammatory response, we only observed activation of the TNF signaling pathway. Through further inspection of the regulated pathways of CBLB stimulated hepatocytes, we could not show CBLB-induced signaling as responsible for the hepatoprotection we observed in vivo. IL-22Rα is constitutively expressed by epithelial cells, including hepatocytes^43^. Treating primary hepatocytes with IL-22 resulted in STAT3 pathway activation; however, the transcriptomic changes were minor. The main pathway affected by IL-22 was the inflammatory response pathway, but only 18 genes were upregulated. This result was unexpected based on the proven activity of STAT3 signaling in vivo in promoting cell survival, proliferation, IRI, and ConA protection^34,35^.

Often transcription factors require transcriptional cooperation in order to initiate gene transcription and interestingly, NF-κB and STAT3 have been largely studied for their transcriptional collaboration^44^. Contrary to the effect they elicit alone, the combination of CBLB + IL-22 induced a strong upregulation of genes involved in the inflammatory response pathway. The transcriptional effect of CBLB + IL-22 contains inhibitors of inflammation, apoptosis, and modulators of redox processes that are regulated following CBLB or IL-22 alone. For example, there was an upregulation of NF-κB pathway inhibitors (*Nfkbia*, *Nfkbid*, *Nfkbiz*, and *Sbno2*) that could be expected to reduce IRI damage, as NF-κB activation was shown to worsen the hepatic damage in IRI and ConA models^45,46^. In addition, the Socs protein family inhibits pathways such as insulin, IFNγ TLR, and p38/JNK signaling also known to exacerbate IRI and ConA injury^17,45–51^. Furthermore, genes involved in redox modulation, such as *Hif1a*, *Hmox1*, *Nos2*, and *Sod2*, were shown to promote resistance in IRI models^52^. Finally, we identified an induction of antiapoptotic genes such as *Tnfaip3* known to promote survival in both hepatic IRI and ConA-induced hepatitis models^53,54^.

As the synergic treatment of CBLB and IL-22 activates a hepatoprotective transcriptional response in hepatocytes in vitro, we evaluated the difference of response of CBLB in IL-22^−/−^ mice. In response to CBLB, IL-22^−/−^ mice showed a decrease of 60% in STAT3 signaling compared to WT mice. Other cytokines induced by CBLB, such as IL-6 and IL-18, are known to activate STAT signaling and might explain the remaining 40% of STAT3 phosphorylation^55,56^. We then confirmed in vivo the liver activation of three of the cytoprotective genes induced in hepatocytes with combined CBLB and IL-22 treatment. Tnfaip3, Hmox1, and Socs3 were strongly upregulated in the livers of WT mice, but this upregulation was absent or diminished in IL-22^−/−^ mice. Moreover, CBLB was not able to provide the same amount of protection in the IL-22^−/−^ mice against ischemia-reperfusion injury. This result is concordant with the known literature on the protective activity of IL-22^34^, TNFAIP3^53^, HMOX1^52^, and SOCS3^46–49^ in ischemia-reperfusion injury.

**Fig. 5:**
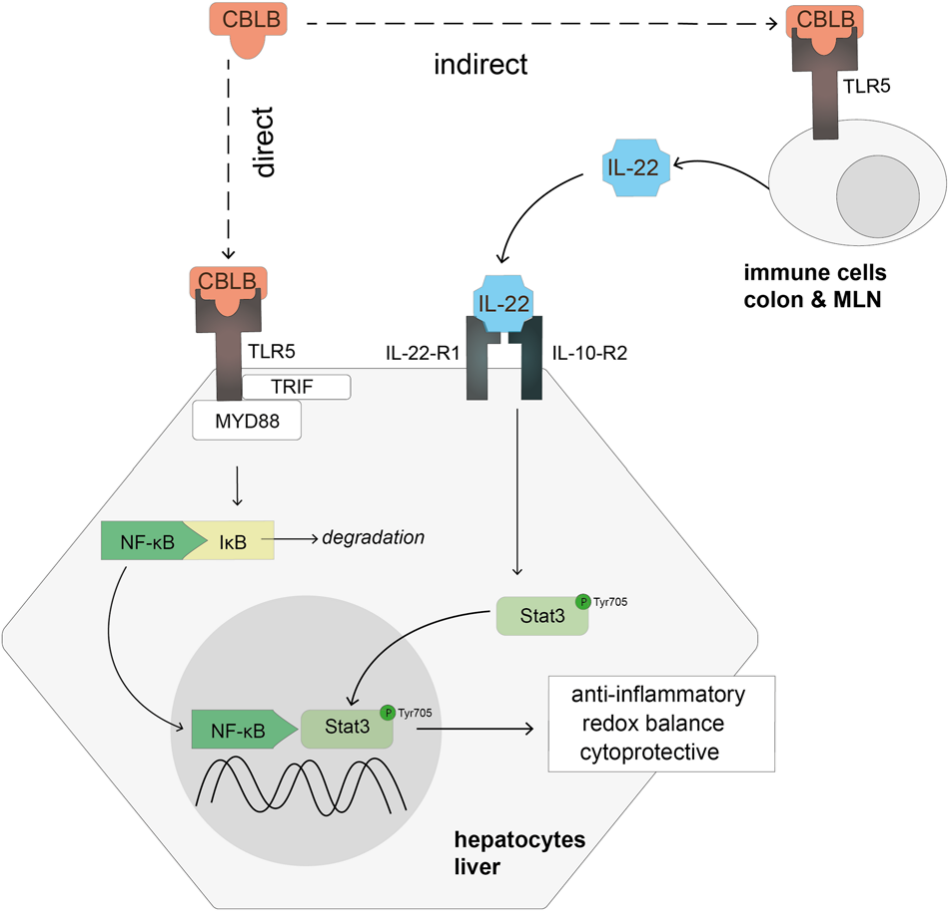
Mechanism of CBLB, a TLR5 agonist, in hepatoprotection. Graphical summary of the direct and indirect action of CBLB on hepatocytes.

Taken together, our data demonstrates that preconditioning the liver with CBLB is protective against acute hepatic injury. CBLB induces a hepatoprotective transcriptional response in hepatocytes through a direct activation of TLR5—NF-κB signaling in hepatocytes, as well as the induction of STAT3 signaling indirectly via the production of IL-22 from the colon and MLN (Fig. 5).

## Supplementary information

supplementary Table 1

supplementary table 2

Supplementary Figure 1

Supplementary Figure 2

Supplementary Figure 3

Supplementary Figure 4

Supplementary Figure 5

Supplementary Figure 6

Supplementary Figure 7

Supplementary Figure 8

supplementary figure legends

## Data Availability

RNA-seq data are available on the GEO database repository (GSE160465; link).
