# Supplementary material for: Synergistic effect of the TLR5 agonist CBLB502 and its downstream effector IL-22 against liver injury
Source: Cell Death Dis. 2021 Apr 6;12(4):366. doi: 10.1038/s41419-021-03654-3 (PMC8024273; doi:10.1038/s41419-021-03654-3)
Supplement: Supplementary file 1 — supplementary Table 1 [file 41419_2021_3654_MOESM1_ESM.docx]

Supplementary table 1: ProcartaPlex immunoassay used (eBioscience).

| Target | Catalogue number |
| --- | --- |
| IL-1α | EPX010-20611-901 |
| IL-1β | EPX010-26002-901 |
| IL-4 | EPX010-20613-901 |
| IL-5 | EPX010-20610-901 |
| IL-6 | EPX010-20603-901 |
| IL-10 | EPX010-20614-901 |
| IL-12p70 | EPX010-26004-901 |
| IL-13 | EPX010-26015-901 |
| IL-15 | EPX010-26023-901 |
| IL-18 | EPX010-20618-901 |
| IL-17a | EPX010-26001-901 |
| IL-22 | EPX010-26022-901 |
| IL-23 | EPX010-26017-901 |
| IFN-α | EPX010-26027-901 |
| IFN-γ | EPX010-20611-901 |
| TNF-α | EPX010-20607-901 |
| CCL2 | EPX010-26005-901 |
| CCL5 | EPX010-20606-901 |
| CXCL1 | EPX010-26031-901 |
| CXCL2 | EPX010-26032-901 |
