# Supplementary material for: Synergistic effect of the TLR5 agonist CBLB502 and its downstream effector IL-22 against liver injury
Source: Cell Death Dis. 2021 Apr 6;12(4):366. doi: 10.1038/s41419-021-03654-3 (PMC8024273; doi:10.1038/s41419-021-03654-3)
Supplement: Supplementary file 2 — supplementary table 2 [file 41419_2021_3654_MOESM2_ESM.docx]

Supplementary table 2: TaqMan probe used (ThermoFisher Scientific).

| Target | Catalogue number |
| --- | --- |
| Tbp | Mm00446971 |
| Il22 | Mm00444241 |
| Tnfaip3 | Mm00437121 |
| Hmox1 | Mm00516004 |
| Socs3 | Mm00545913 |
