# Supplementary figures and images for: Synergistic effect of the TLR5 agonist CBLB502 and its downstream effector IL-22 against liver injury
Source: Cell Death Dis. 2021 Apr 6;12(4):366. doi: 10.1038/s41419-021-03654-3 (PMC8024273; doi:10.1038/s41419-021-03654-3)

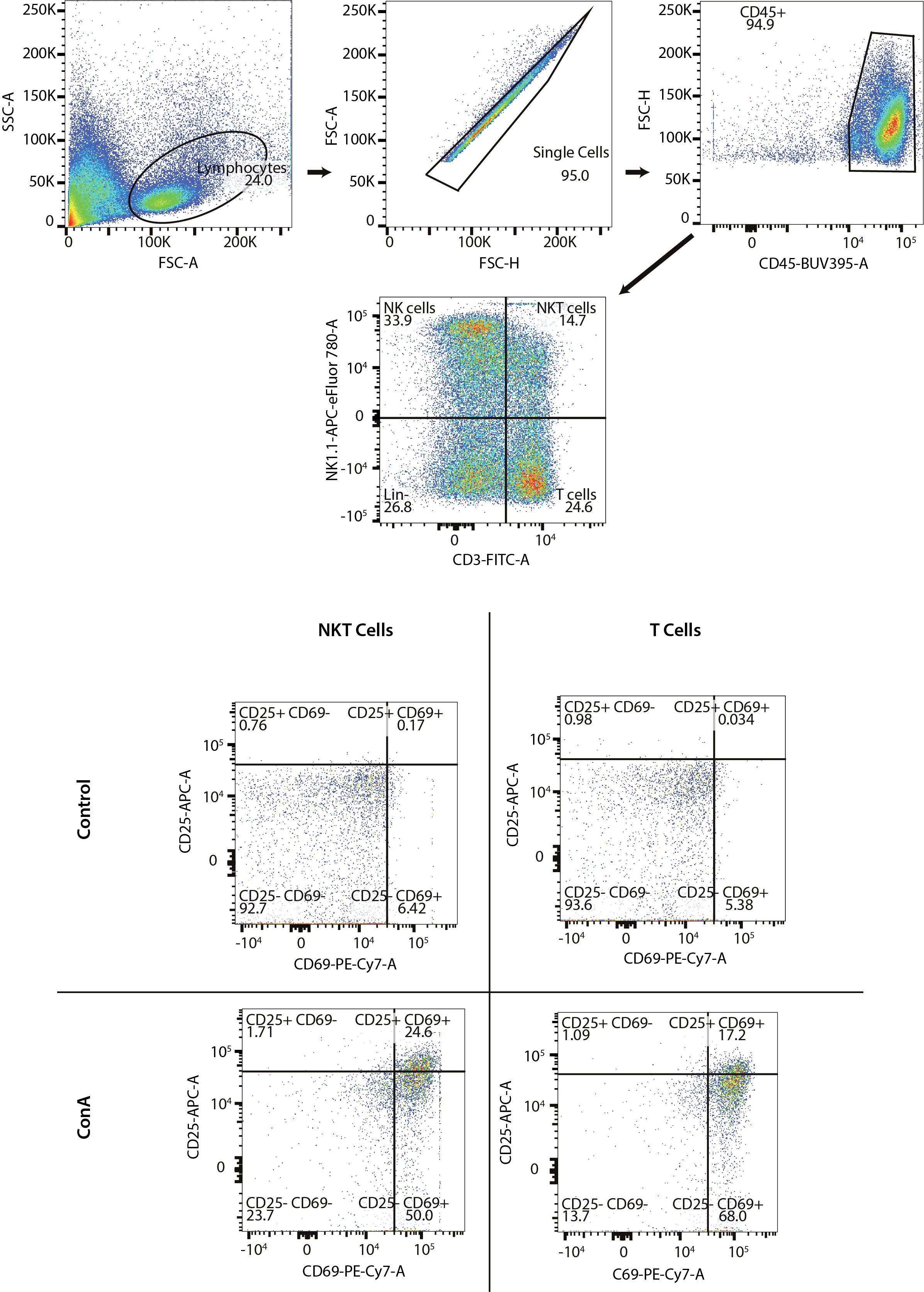

Supplement: Supplementary file 3 — Supplementary Figure 1 [file 41419_2021_3654_MOESM3_ESM.png]

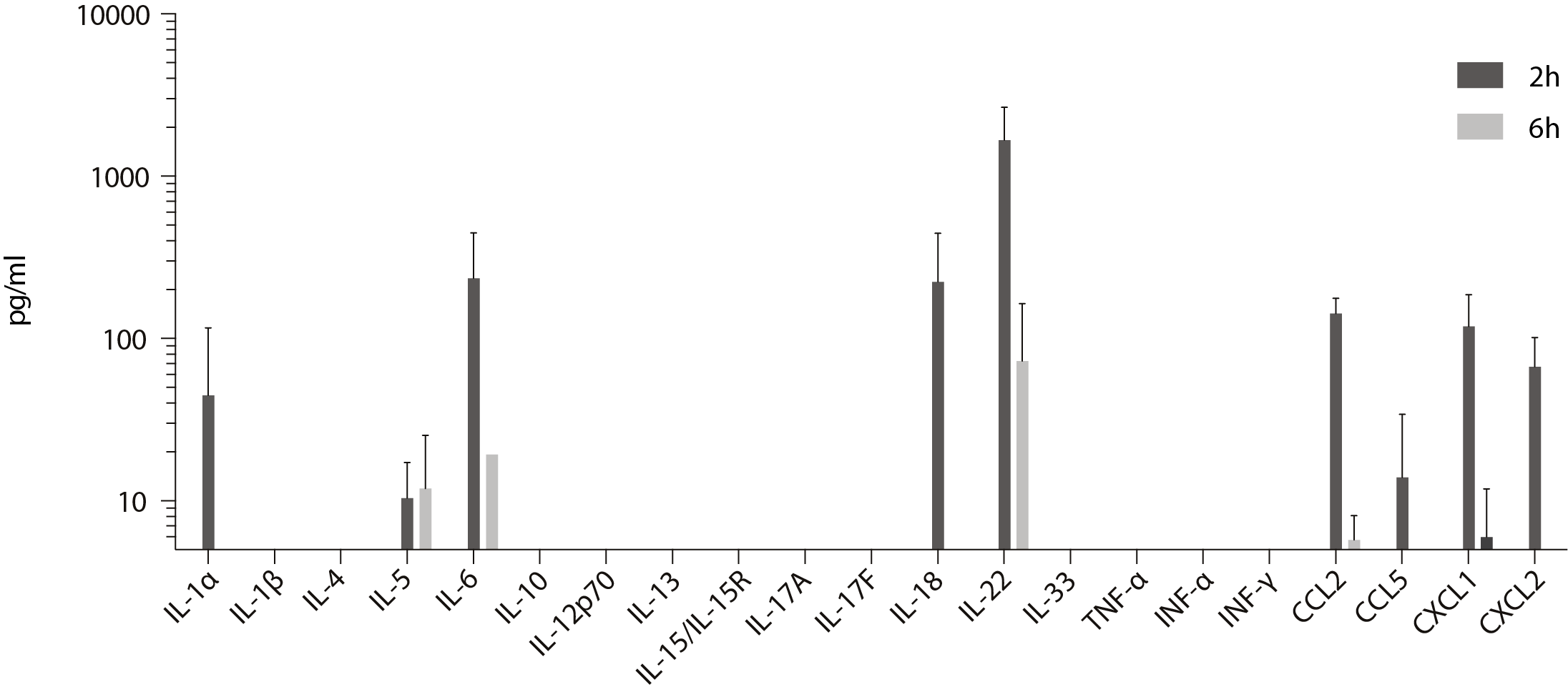

Supplement: Supplementary file 4 — Supplementary Figure 2 [file 41419_2021_3654_MOESM4_ESM.png]

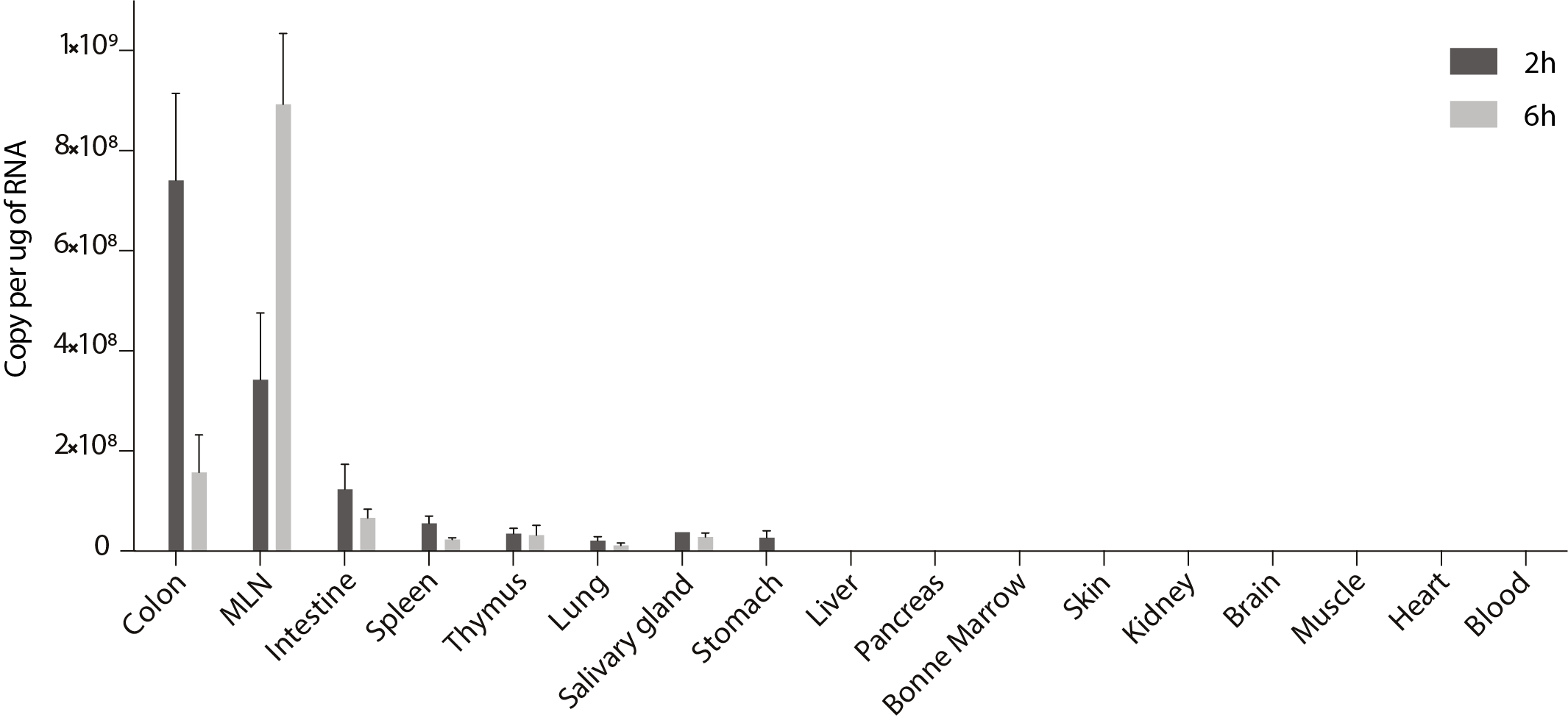

Supplement: Supplementary file 5 — Supplementary Figure 3 [file 41419_2021_3654_MOESM5_ESM.png]

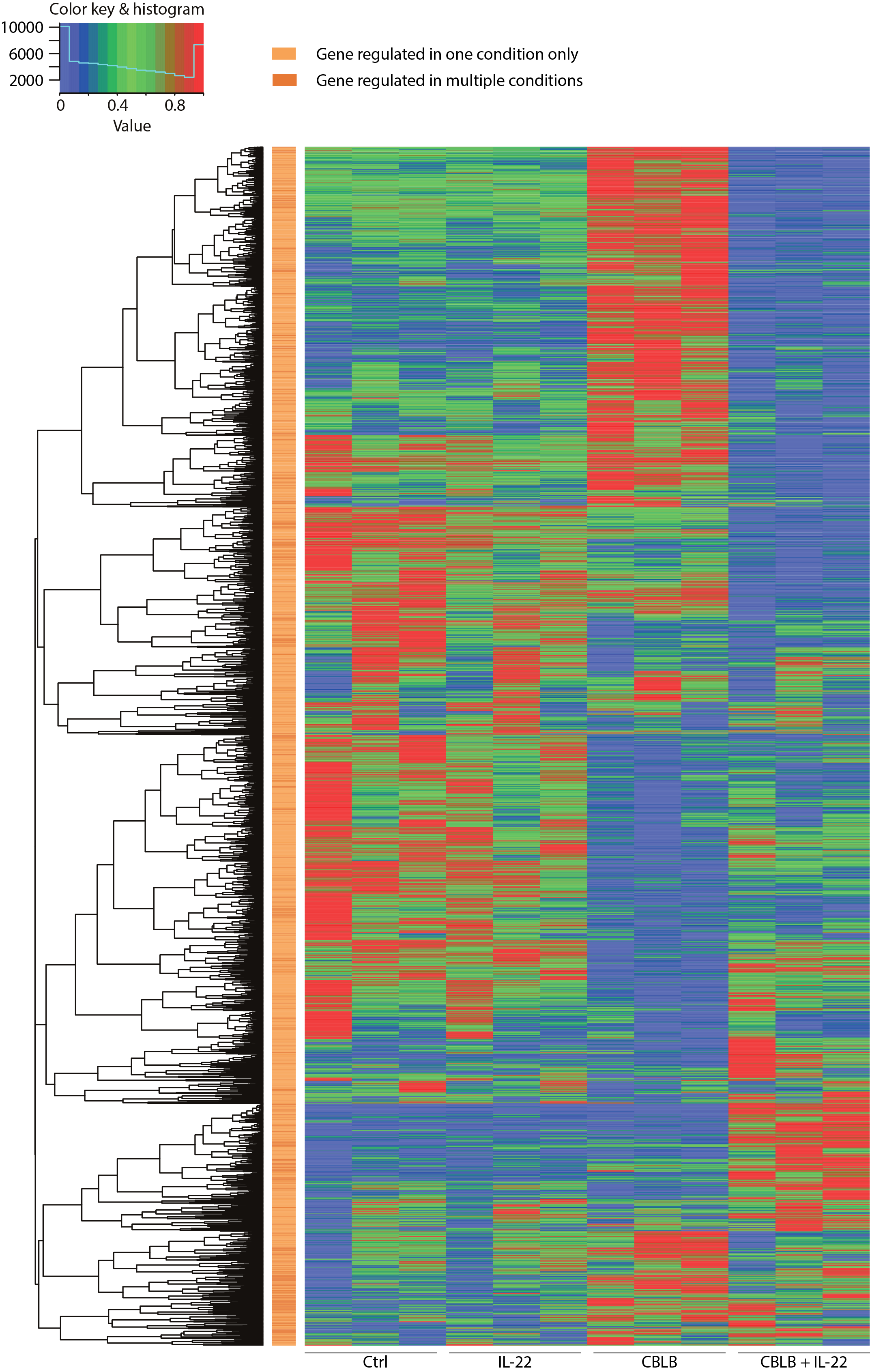

Supplement: Supplementary file 6 — Supplementary Figure 4 [file 41419_2021_3654_MOESM6_ESM.png]

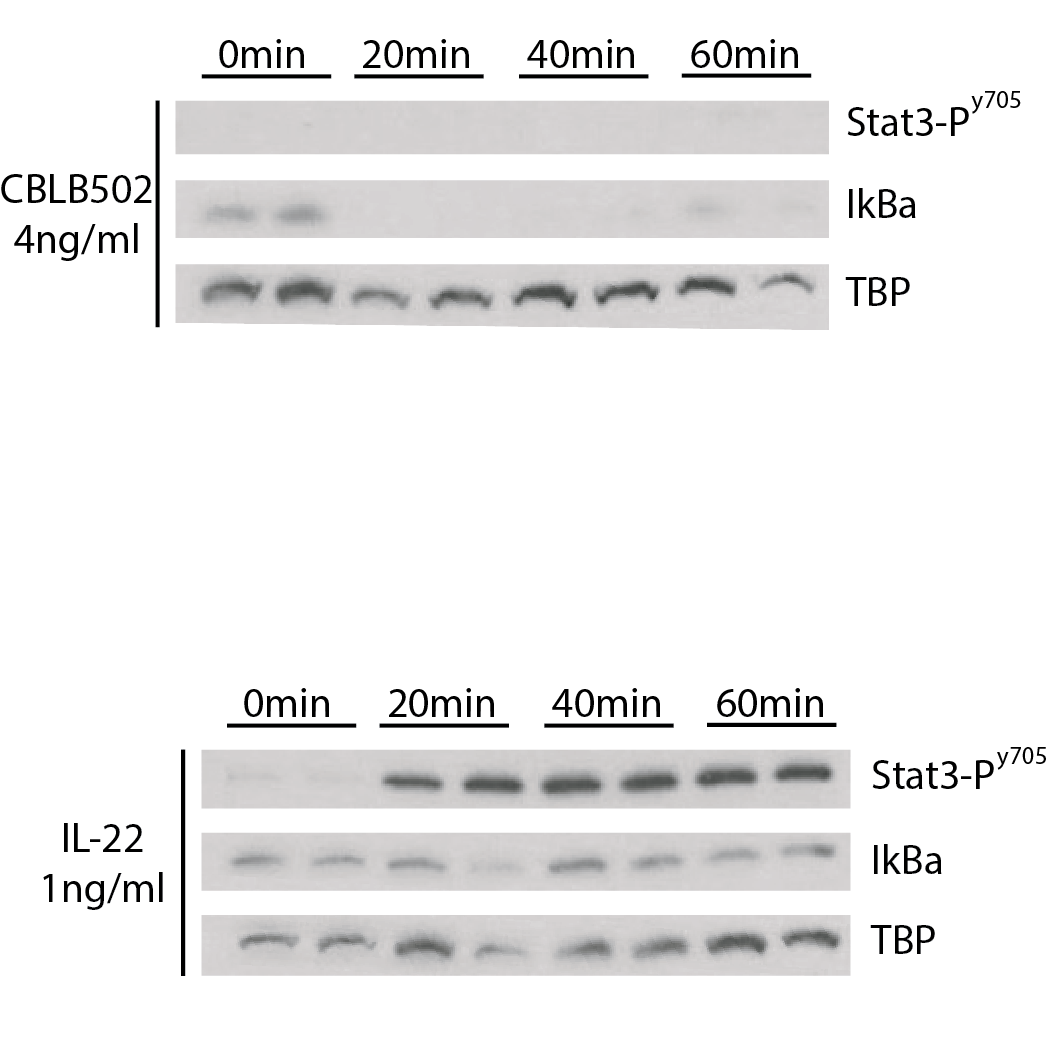

Supplement: Supplementary file 7 — Supplementary Figure 5 [file 41419_2021_3654_MOESM7_ESM.png]

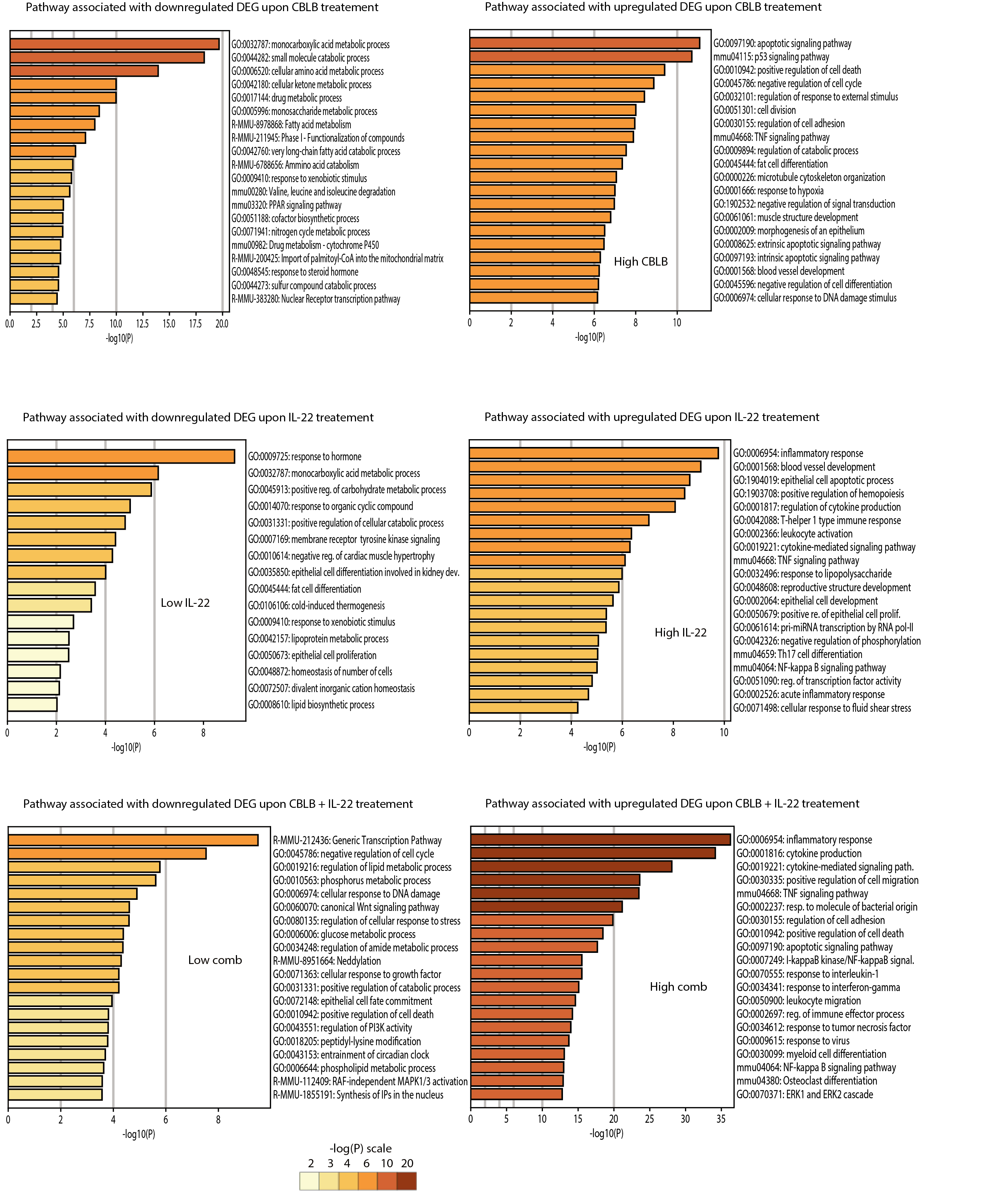

Supplement: Supplementary file 8 — Supplementary Figure 6 [file 41419_2021_3654_MOESM8_ESM.png]

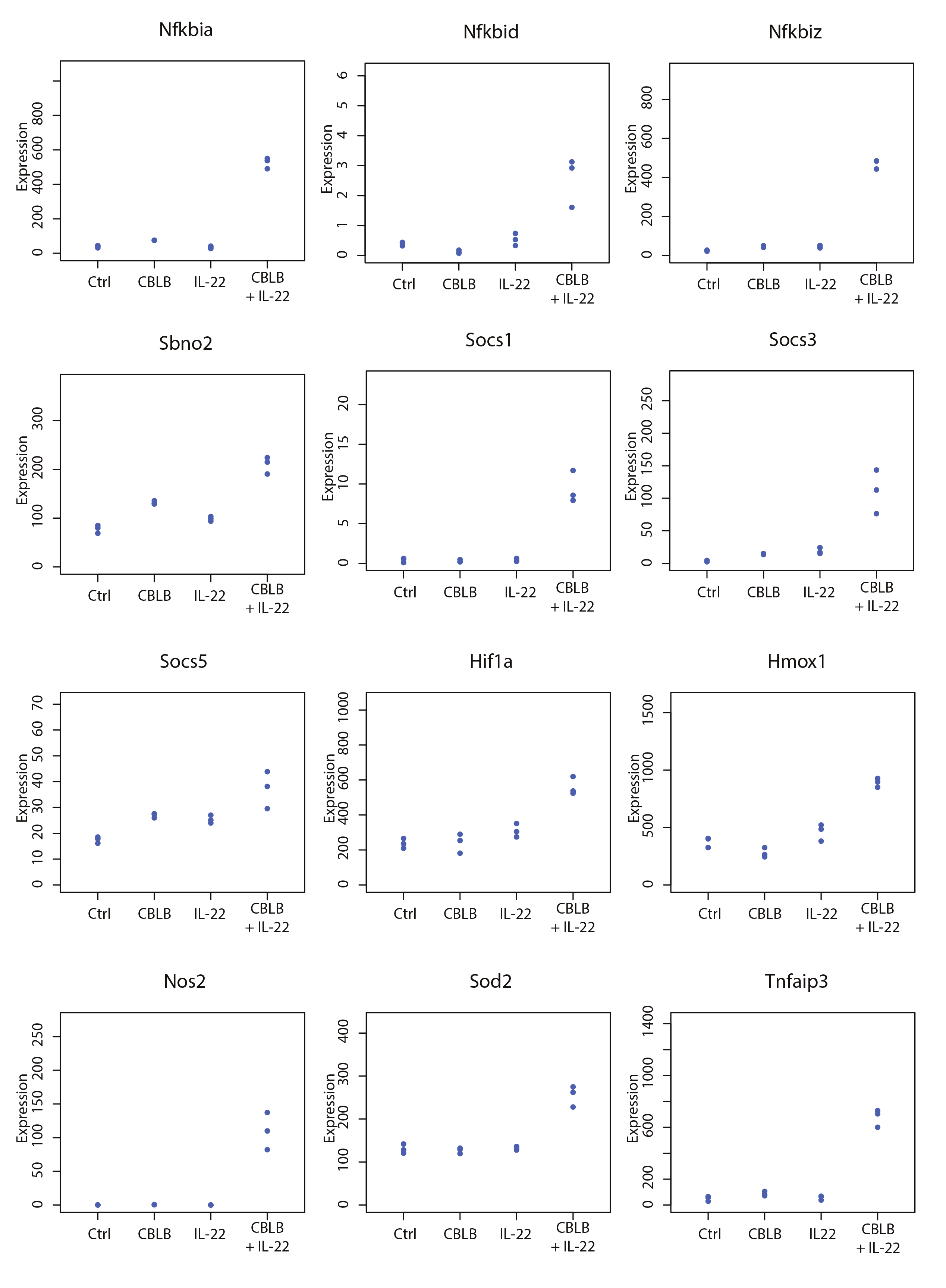

Supplement: Supplementary file 9 — Supplementary Figure 7 [file 41419_2021_3654_MOESM9_ESM.png]

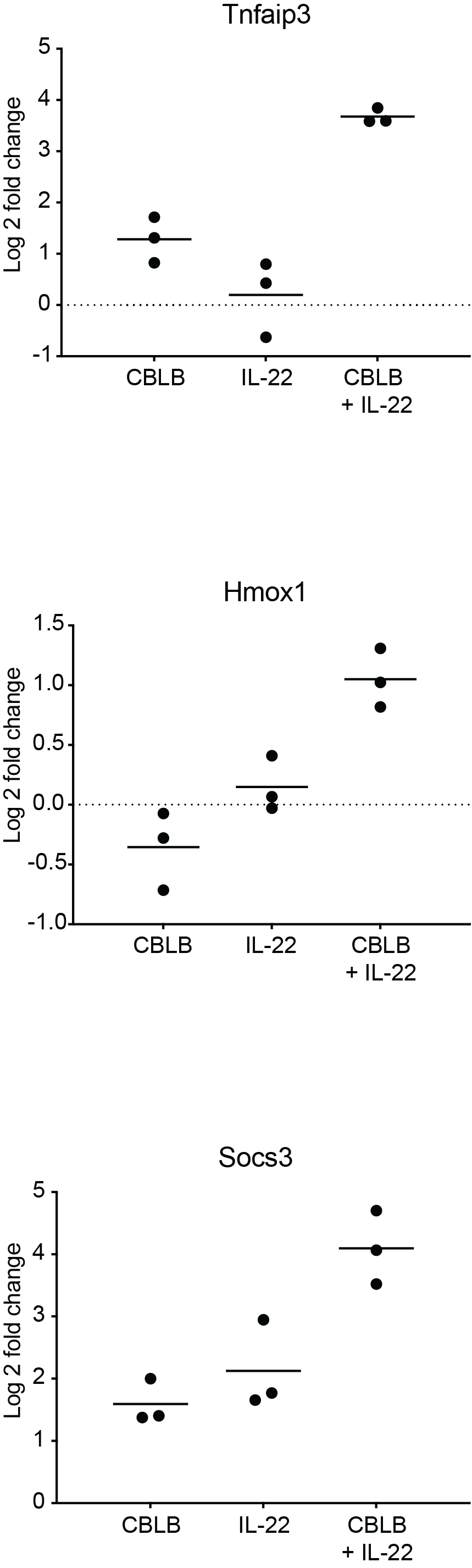

Supplement: Supplementary file 10 — Supplementary Figure 8 [file 41419_2021_3654_MOESM10_ESM.png]
