# Supplementary material for: Synergistic effect of the TLR5 agonist CBLB502 and its downstream effector IL-22 against liver injury
Source: Cell Death Dis. 2021 Apr 6;12(4):366. doi: 10.1038/s41419-021-03654-3 (PMC8024273; doi:10.1038/s41419-021-03654-3)
Supplement: Supplementary file 11 — supplementary figure legends [file 41419_2021_3654_MOESM11_ESM.docx]

Supplementary & Extended Information for

**Synergistic hepatoprotective effect of the TLR5 agonist CBLB502 and its downstream effector IL-22**

**Supplementary Figure 1.** Representative FACS pots showing the gating strategy used, allowing the identification of NKT and T cells and the evaluation of their activation level by looking at CD25 and CD69 markers.

**Supplementary Figure 2**. Serum cytokine profiling two hours and six hours post CBLB-injection.

**Supplementary Figure 3.** IL-22 tissue expression profiling two hours and six hours post injection.

**Supplementary Figure 4.** Heatmap of all the DEG across the three conditions compared to control.

**Supplementary Figure 5.** Western blot analysis of IκBα, STAT3 phosphorylated at tyrosine 705 and TBP (as loading control) of hepatocyte exposed to CBLB and IL-22 for control, twenty, forty and sixty minutes, n=2 replicates per time point.

**Supplementary Figure 6.** Pathway enrichment analysis based on the downregulated and upregulated DEG for the CBLB, IL-22 and CBLB+IL-22 treated hepatocytes.

**Supplementary Figure 7.** Cytoprotective gene expression in read per million.

**Supplementary Figure 8**. qPCR of three cytoprotective genes expressed in mouse hepatocytes in response to CBLB, IL-22 and CBLB+IL22 compared to control.
